# The Prognostic Value of Non-Linear Analysis of Heart Rate Variability in Patients with Congestive Heart Failure—A Pilot Study of Multiscale Entropy

**DOI:** 10.1371/journal.pone.0018699

**Published:** 2011-04-13

**Authors:** Yi-Lwun Ho, Chen Lin, Yen-Hung Lin, Men-Tzung Lo

**Affiliations:** 1 Graduate Institute of Clinical Medicine, National Taiwan University Hospital and National Taiwan University College of Medicine, Taipei, Taiwan; 2 Division of Cardiology, Department of Internal Medicine, National Taiwan University Hospital and National Taiwan University College of Medicine, Taipei, Taiwan; 3 Center for Dynamical Biomarkers and Translational Medicine, National Central University, Chungli, Taiwan; 4 Research Center for Adaptive Data Analysis, National Central University, Taoyuan, Taiwan; 5 Institute of Systems Biology and Bioinformatics, National Central University, Taoyuan, Taiwan; University of Maribor, Slovenia

## Abstract

**Aims:**

The influences of nonstationarity and nonlinearity on heart rate time series can be mathematically qualified or quantified by multiscale entropy (MSE). The aim of this study is to investigate the prognostic value of parameters derived from MSE in the patients with systolic heart failure.

**Methods and Results:**

Patients with systolic heart failure were enrolled in this study. One month after clinical condition being stable, 24-hour Holter electrocardiogram was recording. MSE as well as other standard parameters of heart rate variability (HRV) and detrended fluctuation analysis (DFA) were assessed. A total of 40 heart failure patients with a mea age of 56±16 years were enrolled and followed-up for 684±441 days. There were 25 patients receiving β-blockers treatment. During follow-up period, 6 patients died or received urgent heart transplantation. The short-term exponent of DFA and the slope of MSE between scale 1 to 5 were significantly different between patients with or without β-blockers (p = 0.014 and p = 0.028). Only the area under the MSE curve for scale 6 to 20 (Area_6–20_) showed the strongest predictive power between survival (n = 34) and mortality (n = 6) groups among all the parameters. The value of Area_6–20_ 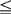
21.2 served as a significant predictor of mortality or heart transplant (p = 0.0014).

**Conclusion:**

The area under the MSE curve for scale 6 to 20 is not relevant to β-blockers and could further warrant independent risk stratification for the prognosis of CHF patients.

## Introduction

Congestive heart failure (CHF) remains to be one of the major cardiovascular disorders in the world [1]. Despite its high expenditure in healthcare budgets [2], the mortality rate of CHF patients can be up to 8 times higher than the age-matched control population [3]. The present treatment protocols of CHF patients, such as administrating angiotensin converting enzyme inhibitors (ACE-I) and β blockers, have been proven to lower the mortality and hospital admission rate [4]. Nevertheless, the residual risk for mortality and morbidity of CHF remains high even under such treatment protocols [5], [6]. Therefore, further novel prognostic predictor is needed to strengthen the treatment strategy in addition to neurohormonal inhibition therapy.

Conventional linear heart rate variability (HRV) analyses, including frequency and time domain analyses, have been reported as prognostic factors for CHF [7], [8]. However, heart rate fluctuations have been recognized as complex behaviors originated from nonlinear processes and often with nonstationary property [9]–[11]. Applying linear algorithms to those seemingly irregular and “patchy” patterns of heart rate fluctuations [12] may cause the intrinsic computational errors of the linear algorithms [11], [13], [14]. Properly use of the analyses based on fractals and chaos theory [15]–[17] to qualify or quantify the characteristics of heart rate time series are suggested to serve as a more reliable index of physiological systems in many clinical studies [10], [11], [18]. As one of such mathematic methods, multiscale entropy (MSE) analysis has focused specifically on characterizing heterogeneous complexity [19]. Such complex structure is “breakdown” (loss of information richness) and points to poor prognosis in CHF patients [19], [20]. We hypothesized that MSE could yield a prognostic marker which was not relevant to neurohormonal inhibition therapy in CHF patients. The aims of this study were 1) to evaluate the influences of β-blockers on parameters derived from MSE; 2) to assess the prognostic significance of parameters derived from MSE for CHF patients.

## Methods

### Study Population

Patients with manifestation of exertional dyspnea, leg edema and systolic heart failure (LVEF<45% by echocardiography) at the National Taiwan University Hospital were enrolled after giving their inform consents. Baseline information, including age, sex, etiologies for heart failure,diabetes mellitus, hypertension, dyslipidemia (total cholesterol >220 mg/dl), and cardiovascular medication use (β-blockers, ACE-I, angiotensin-II receptor blockers, and spironolactone) was reviewed in medical records and charts. Patients with renal dysfunction (defined by creatine≧2.0 mg/dl) were excluded. One month after clinical condition being stable, standard ambulatory 24-hour electrocardiogram (ECG) recorders were placed on all participants. The ECG signals were sampled at 250 Hz and stored in SD memory card for offline analysis on a microcomputer. Subsequently, these patients were followed up and mortality or heart transplantation will be noted as end-point for follow-up. The Ethics Committee of National Taiwan University Hospital approved the study and all patients provided written informed consent.

### Data pre-processing

Each digitalized 24-hour ECG data was annotated by an automated algorithm and the annotated file was then carefully inspected and corrected by technicians for extracting the RR intervals. The ectopic beats (including atrial or ventricular premature beats) were interpolated by its adjacent RR intervals. A four-hour period of RR intervals in daytime (between 9AM–5PM) was selected from each recording to avoid confounding effects on nonlinear or linear analysis caused by different sleep stage or diurnal rhythm [21], [22]. Only subjects consisted of more than 80% of qualified normal sinus beats were included for further analysis (the typical RR-interval tracings and the corresponding recurrent plots for survival and mortality groups (Figure S1) as well as all of the RR-interval data are provided as supplementary materials (Materials S1)).

### Time and frequency domain analysis

Standard deviation of normal RR intervals (SDNN) and percentage of absolute differences in normal RR intervals greater than 50 ms (pNN_50_) were calculated to represent the total variance and vagal modulation of the HR. In addition, the spectrum analysis was carried out in accordance with the recommendations of the European Society of Cardiology and the North American Society of Pacing Electrophysiology [23]. The spectral density of each frequency band-high frequency (HF) (0.15–0.4 Hz), low frequency (LF) (0.04–0.15 Hz), and very low frequency (VLF) (0.003–0.04) were computed by average power spectrum.

### Nonlinear methods

Nonlinear analysis enables the researchers to probe the fundamental characteristics of the signals. However, unwanted inferences such as noise and nonstationarity may introduce spurious features to the signals [24], [25]. The underlying mechanisms of the irregular and unpredicted behavior of the signals can be misinterpreted and the reliability of the results of analysis can be compromised. Two methods had been chosen for their ability to evaluate the main properties of the signals [14], [26].

### Detrended fluctuation analysis (DFA)

DFA is a modified root-mean-square analysis used to evaluate the fractal correlation beneath the heart rate fluctuation originated from the interacted regulatory mechanisms. The algorithm has been described in detail elsewhere [14]. To briefly introduce this method, at first, it eliminates the environmental inferences by removing the linear-fitted “local” trend over different time scales (“box sizes”) in an integrated time series. Next, the root-mean-square fluctuation of this integrated and detrended time series is calculated. This procedure is repeated over different time scales and then the slope of the curve (α exponent) can be estimated on the log-log plot of fluctuations versus box sizes.

In addition, a crossover phenomenon of α exponent in heart rate dynamics between short (4–11 beats) and long (11–64 beats) time scales has been proposed. The short-term (α_1_) as well as long-term (α_2_) fractal correlation exponents were calculated to provide better understanding of the fractal correlation property in physiological system [14].

### MSE analysis

**Figure 1:**
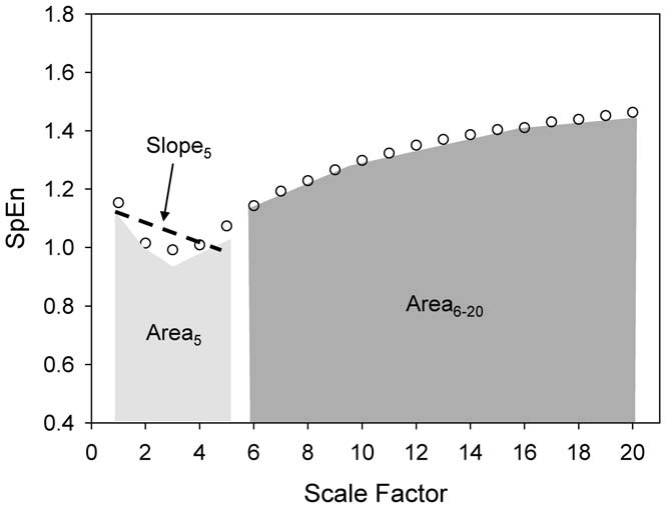
Demonstrative graph of MSE derived parameters. The profile of MSE can be assessed by a) its linear-fitted slope between certain scales which represent the complexity behaviors of the signals. The negative slope may indicate a random-like structure over certain timescale. B) the area under curve between certain scales that may represent its quantitative feature of the underlying physiological mechanisms in certain time scales (ex. area under scale1∼5 may respond to the ability of respiratory sinus arrhythmia).

Instead of simply using single time scale to estimate the complex pattern (irregularity) of a time series, MSE extended this concept to evaluate the complexity of physiological signals on multiple time scales. It comprises of two steps: 1) coarse-graining the signals into different time scales; 2) quantify the degree of irregularity in each coarse-grained time series using sample entropy (SpEn) [27]. Finally, the entropy is calculated as a function of scale, providing a measure of information richness embedded in different time scales. In addition, it has shown that different features of small and large scales in different groups of subjects may assist the clinical categorization [19] and thus three different parameters were derived from the MSE profile: the summations of quantitative values of scale 1–5 (Area_5_) or scale 6–20 (Area_6–20_) which represent the complexity exhibit in short and long time scales, respectively; and the linear-fitted slope of the first 5 scales (Slope_5_) (Figure 1). Although MSE was successfully applied in physiological signals [18], [19], [24], nonstationary artifacts especially trends can compromise the estimation of entropy-based analysis by increasing the standard deviation of the data. Hence, detrending process was used to attenuate the spurious influence caused by nonstationarity [24].

In this study, the empirical mode decomposition (EMD) method was adopted as an adaptive filter to eliminate the oscillations slower than VLF range in the original R-R interval signals [28]. The data subsequently evaluated by the MSE analysis after detrending. This algorithm, instead of removing trend with *a priori* mathematical formulas such as linear or polynomial functions [14], [29], could evaluate the hidden dynamics of heart beat fluctuations better [9], [10], [29].

### Statistical analysis

For the independence of different nominal variables between groups, the chi-square test or Fisher exact test were performed. The continuous variables were represented as mean value ± SD and the normality of those variables was evaluated by using the Shapiro-Wilk test. Then, the Mann-Whitney U test or Student's t test was applied to the between-group comparison accordingly while the Wilcoxon sign test or Student's paired t test was calculated for the intra-group comparison. The receiver operating characteristic curve (ROC) was constructed by the sensitivity and specificity of the continuous variable in predicting the end-point. Area under the ROC curve (AUCs) gave an estimate of the overall discriminate ability. Furthermore, the most predictive indexes will be selected to seek the optimal cut point within the 30^th^ to 70^th^ percentile for all patients in 5^th^-percentile step. The maximal hazards ratio and independent correlation of variables with event status (mortality) was determined by Cox regression analysis. Then, Kaplan-Meier event probability curves and log rank analysis of the dichotomized groups were obtained. The statistical significance was set at *p*<0.05.

## Results

### Characteristics of Patients

A total of 40 heart failure patients (30 males and 10 females) with a mean age of 56±16 years were enrolled and followed-up for 684±441 days. Twenty-five patients received β-blockers (either carvedilol or metoprolol). Carvedilol was titrated from 3.25 mg per day and metoprolol was titrated from 12.5 mg per day to maximal tolerable doses. Six patients died or received heart transplantation during follow-up period of this study. The demographic and clinical data were showed in Table 1. No clinical variable was significantly different between these two groups (with or without β-blocker therapy).

**Table 1:** The clinical characteristics between patients with and without using β-blockers.

Patient characteristics	β-blockers(−)(n = 15)	β-blockers (+)(n = 25)	*p* value
Age (years)	61.7±14.7	51.8±13.5	*p* = 0.073
Male/Female	9/6	21/4	*p* = 0.158
Heart rate (bpm)	86±13	87±15	*p* = 0.793
LVEF(%)	33±12	35±14	*p* = 0.684
Creatinine	1.07±0.25	1.18±0.35	*p* = 0.280
Fasting sugar (mg/dl)	113±47	122±47	*p* = 0.573
Triglyceride (mg/dl)	129±85	195±177	*p* = 0.189
Cholesterol (mg/dl)	185±35	185±65	*p* = 0.988
Hemoglobin (g/dl)	13.6±1.9	13.4±2.3	*p* = 0.712
Uric acid (mg/dl)	8.9±3.2	7.1±2.6	*p* = 0.138
NYHA functional class			*p* = 0.660
**I**	2	4	
**II**	8	10	
**III**	5	9	
**IV**	0	2	
Body mass index	25.4±5.2	25.6±5.3	*p* = 0.929
Etiology of heart failure			*p* = 0.924
Coronary artery disease	6	11	
Non-coronary artery Diseases	9	14	
Hypertension	4	11	
Diabetes mellitus	6	10	
Medication			*p* = 0.845
ACE-I/ARB	12	19	
Loop diuretics	10	18	
Digoxin	9	10	
Spironolactone	6	7	

A total of 40 heart failure patients (30 males and 10 females) were enrolled in this study. No clinical variable was significantly different between the patients with or without β-blocker therapy.

NYHA = New York Heart Association; ACE-I = angiotensin converting enzyme inhibitor; ARB = angiotensin receptor blocker.

### Effect of β-blockers on autonomic activities and its fractal properties

While the conventional HRV measurements showed no significant different between these two groups, DFAα_1_ showed significantly higher values (*p* = 0.014) in patients with β-blocker treatment (Table 2).

**Table 2:** Effect of the β-blockers on the autonomic activities, fractal properties and MSE.

	β-blockers(−)(n = 15)	β-blockers(+)(n = 25)	*p* value
Time domain analysis			
SDNN	53.2±17.8	49.1±26.9	***p*** = .332
pNN_50_	0.91±0.85	0.76±1.13	***p*** = .100
Frequency domain analysis			
HF	31.43±20.91	27.9±35.3	***p*** = .088
LF	56.3±43.0	90.9±102.9	***p*** = .659
VLF	484.8±321.7	563.0±462.9	***p*** = .956
Detrended fluctuation analysis			
α_1_	0.91±0.22	1.10±0.34	***p*** = .014
α_2_	1.27±0.09	1.26±0.18	***p*** = .679
Multiscale entropy			
Slope_5_	−0.02±0.07	0.03±0.08	***p*** = .028
Area**_5_**	5.3±1.2	5.5±1.1	***p*** = .719
Area**_6–20_**	13.1±3.0	14.3±2.7	***p*** = .211

While the conventional HRV measurements showed no significant different between these two groups, nonlinear indices, DFA_α1_ and the value of Slope_5_, were significantly lower in patients without β-blocker therapy.

Slope_5_ = the linear-fitted slope of the first 5 scales, Area**_5_** = the summations of quantitative values of scale 1–5, Area**_6–20_** = the summations of quantitative values of scale 6–20.

### Effect of β-blockers on the dynamical complexity assessed by the MSE analysis

There was no significant difference when any single sample entropy value of scales 1 to 20 was compared between patient groups with or without β-blockers. However, the value of Slope_5_ was not only significantly lower (*p* = 0.028) in patients without β-blocker therapy but also exhibit a negative value (Table 2).

### Application of MSE in prognostic prediction

**Figure 2:**
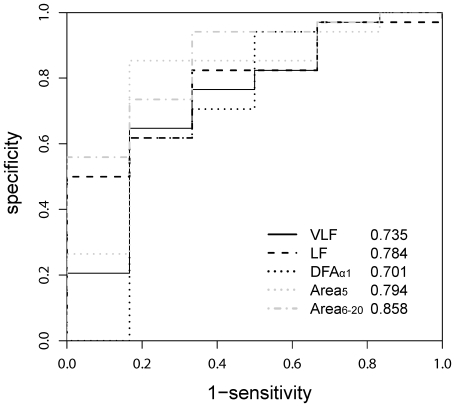
Very-low-frequency component (VLF; black, solid line), low-frequency component (LF; black, dashed line), short-term fractal exponent (DFAα_1_; black, dotted line), the summations of quantitative values of scale 1–5 (Area_5_; grey, dashed line), and the summations of quantitative values of scale 6–20 (Area_6–20_; grey, dash-dotted line) receiver operating characteristic (ROC) curves. The area under each ROC curves (AUC) was calculated for each parameters. The AUCs were 0.735 for VLF, 0.784 for LF, 0.701 for DFAα_1_, 0.794 for Area_5_, and 0.858 for Area_6–20_.

**Figure 3:**
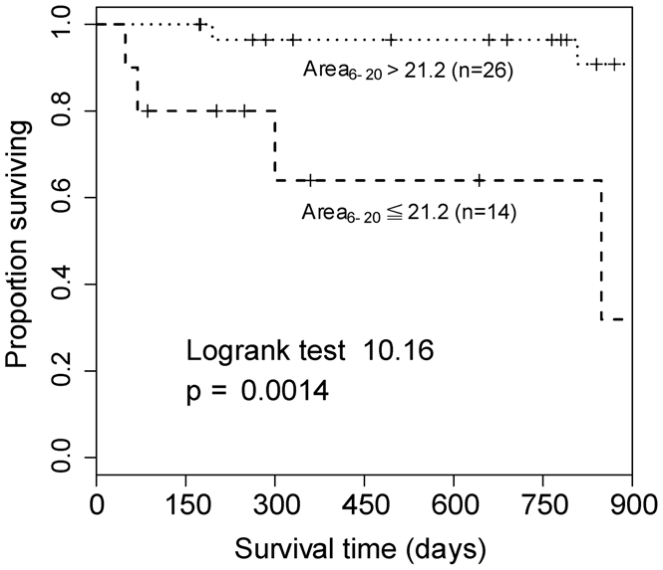
Using MSE Area_6–20_ 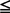
21.2 as a clinical predictor, significant difference in survival was noted from the Kaplan-Meier survival curve (*P = 0.0014*).

Among all parameters, Area_5_, Area_6–20_, and LF were significantly lower (*p* = 0.027, *p* = 0.021, and *p* = 0.004 respectively) in the mortality group. (Table 3) Moreover, the ROC of the previously proposed predictors (LF, VLF, and DFAα_1_) and the new derived parameters (Area_5_ and Area_6–20_) were depicted in Figure 2. Area_6–20_ (AUC:0.858±0.075) showed the best overall discriminative power than LF (AUC: 0.784±0.087), VLF (AUC:0.735±0.117), DFAα_1_ (AUC:0.71±0.145) and Area_5_ (AUC:0.794±0.108) in mortality or heart transplant prediction. Therefore, Area_6–20_ was adopted to perform the analysis of Kaplan-Meier survival curves. The value of Area_6–20_ 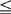
21.2 was a significant predictor of mortality or heart transplant (p = 0.0014). (Figure 3)

**Table 3:** Prognostic value of parameters of HRV.

	Survival group(n = 34)	Mortality group(n = 6)	*p* value
Time domain analysis			
SDNN	52.8±23.5	38.4±22.9	***p*** = .225
pNN_50_	0.88±1.09	0.46±0.37	***p*** = .939
Frequency domain analysis			
HF	32.1±32.0	12.6±9.9	***p*** = .092
LF	87.6±90.1	23.3±22.6	***p*** = .027
VLF	575.4±420.4	297.4±290.5	***p*** = .071
Detrended fluctuation analysis			
α_1_	1.06±0.27	0.84±0.45	***p*** = .127
α_2_	1.28±0.13	1.15±0.23	***p*** = .239
Multiscale entropy			
Slope_5_	0.02±0.07	−0.03±0.11	***p*** = .197
Area**_5_**	5.6±1.0	4.5±1.1	***P*** = .021
Area**_6–20_**	22.3±3.5	16.3±4.5	***p*** = .004

Among all parameters, Area_5_, Area_6–20_, and LF were significantly lower (*p* = 0.027, *p* = 0.021, and *p* = 0.004 respectively) in the mortality group and those indices may potentially serve as outcome predictors.

Slope_5_ = the linear-fitted slope of the first 5 scales, Area**_5_** = the summations of quantitative values of scale 1–5, Area**_6–20_** = the summations of quantitative values of scale 6–20.

## Discussion

Although the effects of β-blockers on HRV indices have been extensively studied [30]–[32], the effects of β-blockers on MSE are still unclear. The present study was the first study to assess the relationship between β-blockers and MSE in CHF patients. The main findings of this preliminary study were that the β-blocker therapy may change the short-term complexity (the slope of MSE between scale 1 to 5). But the most significant predictor of mortality or heart transplantation was the long-term complexity (the area under the MSE curve for scale 6 to 20). Therefore, we found an alternative prognostic predictor of CHF in addition to neurohormonal inhibition therapy by assessing the nonlinear characteristics of the heart rate fluctuations.

### Effect of β-blocker therapy on linear and nonlinear properties of HRV

All linear HRV measurement showed no difference between patients with or without β-blocker treatment in the present study. Other researchers have proposed that the restoration of autonomic function can be assessed by HRV indices [30], [31], [33]. The discrepancy between our finding and previous studies could be due to several factors. The first, the β-blockers were titrated according to patients' tolerance in our study. Therefore, the duration and dosage of β-blockers were variable. The second, the ECG was recorded 1 month after clinical condition being stable in our study. However, significant changes of most linear HRV parameters are found after 12 weeks of treatments. The short-term fractal scaling correlation index, DFAα_1_, was markedly higher in β-blocker group. The reversal of DFAα_1_ is also reported after administrating β-blocker in patients with CHF [33]. Our data showed similar results.

The new nonlinear method, MSE, allows us to evaluate the information richness in heart beat time series over different time scales. Although the underlying mechanisms responsible for the quantitative feature of the MSE in different time scales are still unclear, previous studies have shown that the complexity decreased significantly during aging and further deteriorated in patients with CHF [19]. We applied three different parameters, Area_5_, Area_6–20_, and Slope_5_, to compare patients with or without β-blocker treatment. The summation of entropy values at different time scales may give the quantitative estimation of information richness over certain time scales. That is, Area_5_ and Area_6–20_ can probe the complexity structure of the heart rate dynamics in short (e.g., 1 to 5 heart beats) and longer (e.g., 6 to 20 heart beats) time scales, respectively. The Slope_5_ also outlined the structure of heart rate dynamics in short time scale [19]. The negative value of Slope_5_ indicates random-like patterns in short time scales. Therefore, the significant difference between β-blocker and non β-blocker groups might due to dysfunction of the short-term regulatory mechanisms coincided with the results assessed by DFAα_1_.

### Complexity analysis as a prognosis predictor

Applying Fourier-based method to nonlinear or nonstationary signals may result in inaccurate estimations [9], [29] and compromise the sensitivity of linear HRV measurements. Recently, DFAα_1_ has been proposed to be a better predictor for CHF patients [34]. In the present study, SDNN and DFAα1 failed to predict the prognosis of CHF patients. Makikallio *et al.* noticed that HRV indexes such as SDNN is less sensitive in CHF patient with NYHA>III [35]. Sixteen patients (40%) were classified as NYHA III–IV in our study and five out of them were in mortality group. Moreover, administration of beta-blockade has shown a reversal effect in either linear and nonlinear parameters such as SDNN, HF, LF and DFAα_1_ [30], [32], [33]. Half of the patients with poor outcome were treated with beta blocker which may potentially influence the parameters. SDNN and DFAα_1_ may be, therefore, insensitive to predict their prognosis. Area_5_ and Area_6–20_ derived from MSE were markedly lower in the mortality group. This phenomenon was in agreement with those found by Costa *et al.* in MUSIC study [19]. Although the underlying mechanisms were still unclear, this preliminary study provided a new insight for the prognosis of CHF by probing the dynamical complexity on the system level. It could potentially offer an alternative marker for the outcome of CHF in addition to neurohormonal inhibition therapy.

### Limitations of study

First, our study had small sample size and no placebo-controlled group. Second, all ECG data were recorded in normal “free-running” conditions with possible confounding factors (e.g., physical activities, different breathing patterns, and so on). The additive or dynamical noises may affect the properties of the signals. Although we examined and interpreted the results of the analysis cautiously and the differences characteristics of the patients were unlikely due to the noise, we did not assess the features or level of noise for more detailed information that may benefit the exploration of the underlying deterministic rules. Finally, some parameters related to possible physiological mechanisms of MSE were not collected, such as baroreflex sensitivity, catecholamine levels, and chemoreflex activities.

In conclusions, the area under the MSE curve for scale 6 to 20 is not relevant to β-blockers and could further warrant independent risk stratification for the prognosis of CHF patients.

## Supporting Information

Figure S1 **The 4-hour time tracings of RR intervals of survived patient (A) and patient who died after 1 year (B) and the return map traits of three-dimensional RR time series reconstruction (x-axis for RRn, y-axis for RR_n+1_ and z-axis for RR_n+2_) of survived patient (C) and expired patients (D).** Note that the trait of the map in survival patient was similar to that in expired patient.
(TIFF)

Materials S1 **The RR intervals of each patient was output in a one-column Ascii file and categorized into two groups according to their outcomes after 2.5 years follow up.** All of the files were packed into a compressed RAR file.
(RAR)

## References

[1] Wang TJ, Evans JC, Benjamin EJ, Levy D, LeRoy EC (2003). Natural history of asymptomatic left ventricular systolic dysfunction in the community.. Circulation.

[2] Stewart S, Jenkins A, Buchan S, McGuire A, Capewell S (2002). The current cost of heart failure to the National Health Service in the UK.. Eur J Heart Fail.

[3] van Jaarsveld CH, Ranchor AV, Kempen GI, Coyne JC, van Veldhuisen DJ (2006). Epidemiology of heart failure in a community-based study of subjects aged > or  = 57 years: incidence and long-term survival.. Eur J Heart Fail.

[4] Flather MD, Yusuf S, Kober L, Pfeffer M, Hall A (2000). Long-term ACE-inhibitor therapy in patients with heart failure or left-ventricular dysfunction: a systematic overview of data from individual patients. ACE-Inhibitor Myocardial Infarction Collaborative Group.. Lancet.

[5] Dahlof B, Devereux RB, Kjeldsen SE, Julius S, Beevers G (2002). Cardiovascular morbidity and mortality in the Losartan Intervention For Endpoint reduction in hypertension study (LIFE): a randomised trial against atenolol.. Lancet.

[6] Cohn JN, Tognoni G (2001). A randomized trial of the angiotensin-receptor blocker valsartan in chronic heart failure.. N Engl J Med.

[7] Fauchier L, Babuty D, Cosnay P, Fauchier JP (1999). Prognostic value of heart rate variability for sudden death and major arrhythmic events in patients with idiopathic dilated cardiomyopathy.. J Am Coll Cardiol.

[8] La Rovere MT, Pinna GD, Maestri R, Mortara A, Capomolla S (2003). Short-term heart rate variability strongly predicts sudden cardiac death in chronic heart failure patients.. Circulation.

[9] Lo MT, Tsai PH, Lin PF, Lin C, Hsin YL (2009). The nonlinear and nonstationary properties in EEG signals: probing the complex fluctuations by Hilbert Huang Transform.. Adv Adapt Data Anal.

[10] Peng CK, Costa M, Goldberger AL (2009). Adaptive data analysis of complex fluctuations in physiologic time series.. Adv Adapt Data Anal.

[11] Goldberger AL, Amaral LA, Hausdorff JM, Ivanov PC, Peng CK (2002). Fractal dynamics in physiology: alterations with disease and aging.. Proc Natl Acad Sci U S A.

[12] Buchman TG (2002). The community of the self.. Nature.

[13] Lombardi F (2000). Chaos theory, heart rate variability, and arrhythmic mortality.. Circulation.

[14] Peng CK, Havlin S, Stanley HE, Goldberger AL (1995). Quantification of scaling exponents and crossover phenomena in nonstationary heartbeat time series.. Chaos.

[15] Jagric T, Marhl M, Stajer D, Kocjancic ST, Jagric T (2007). Irregularity test for very short electrocardiogram (ECG) signals as a method for predicting a successful defibrillation in patients with ventricular fibrillation.. Transl Res.

[16] Perc M (2005). Nonlinear time series analysis of the human electrocardiogram.. European Journal of Physics.

[17] Perc M (2005). The dynamics of human gait.. European Journal of Physics.

[18] Yuan HK, Lin C, Tsai PH, Chang FC, Lin KP (2011). Acute increase of complexity in the neurocardiovascular dynamics following carotid stenting.. Acta Neurol Scand.

[19] Costa M, Goldberger AL, Peng CK (2005). Multiscale entropy analysis of biological signals.. Phys Rev E Stat Nonlin Soft Matter Phys.

[20] Costa M, Cygankiewicz I, Zareba W, Bayes de Luna A, Goldberger AL (2006). Multiscale Complexity Analysis of Heart Rate Dynamics in Heart Failure: Preliminary Findings from the MUSIC Study.. Comput Cardiol.

[21] Pikkujamsa SM, Makikallio TH, Sourander LB, Raiha IJ, Puukka P (1999). Cardiac interbeat interval dynamics from childhood to senescence: comparison of conventional and new measures based on fractals and chaos theory.. Circulation.

[22] Vanoli E, Adamson PB, Ba L, Pinna GD, Lazzara R (1995). Heart rate variability during specific sleep stages. A comparison of healthy subjects with patients after myocardial infarction.. Circulation.

[23] (1996). Heart rate variability: standards of measurement, physiological interpretation and clinical use. Task Force of the European Society of Cardiology and the North American Society of Pacing and Electrophysiology.. Circulation.

[24] Costa M, Priplata AA, Lipsitz LA, Wu Z, Huang NE (2007). Noise and poise: Enhancement of postural complexity in the elderly with a stochastic-resonance-based therapy.. Europhys Lett.

[25] Kantz H, Schreiber T (2004). Nonlinear Time Series Analysis.

[26] Costa M, Goldberger AL, Peng CK (2002). Multiscale entropy analysis of complex physiologic time series.. Phys Rev Lett.

[27] Richman JS, Moorman JR (2000). Physiological time-series analysis using approximate entropy and sample entropy.. Am J Physiol Heart Circ Physiol.

[28] Wu Z, Huang NE, Long SR, Peng CK (2007). On the trend, detrending, and variability of nonlinear and nonstationary time series.. Proc Natl Acad Sci U S A.

[29] Lo MT, Novak V, Peng CK, Liu Y, Hu K (2009). Nonlinear phase interaction between nonstationary signals: a comparison study of methods based on Hilbert-Huang and Fourier transforms.. Phys Rev E Stat Nonlin Soft Matter Phys.

[30] Sanderson JE, Chan SK, Yip G, Yeung LY, Chan KW (1999). Beta-blockade in heart failure: a comparison of carvedilol with metoprolol.. J Am Coll Cardiol.

[31] Mortara A, La Rovere MT, Pinna GD, Maestri R, Capomolla S (2000). Nonselective beta-adrenergic blocking agent, carvedilol, improves arterial baroflex gain and heart rate variability in patients with stable chronic heart failure.. J Am Coll Cardiol.

[32] Sanderson JE, Yeung LY, Chan S, Tomlinson B, Kay R (1999). Effect of beta-blockade on baroreceptor and autonomic function in heart failure.. Clin Sci (Lond).

[33] Lin LY, Lin JL, Du CC, Lai LP, Tseng YZ (2001). Reversal of deteriorated fractal behavior of heart rate variability by beta-blocker therapy in patients with advanced congestive heart failure.. J Cardiovasc Electrophysiol.

[34] Ho KK, Moody GB, Peng CK, Mietus JE, Larson MG (1997). Predicting survival in heart failure case and control subjects by use of fully automated methods for deriving nonlinear and conventional indices of heart rate dynamics.. Circulation.

[35] Makikallio TH, Hoiber S, Kober L, Torp-Pedersen C, Peng CK (1999). Fractal analysis of heart rate dynamics as a predictor of mortality in patients with depressed left ventricular function after acute myocardial infarction. TRACE Investigators. TRAndolapril Cardiac Evaluation.. Am J Cardiol.

